# Detection of Water on Road Surface with Acoustic Vector Sensor

**DOI:** 10.3390/s23218878

**Published:** 2023-11-01

**Authors:** Józef Kotus, Grzegorz Szwoch

**Affiliations:** Department of Multimedia Systems, Faculty of Electronics, Telecommunication and Informatics, Gdansk University of Technology, Narutowicza 11/12, 80-233 Gdańsk, Poland; grzszwoc@pg.edu.pl

**Keywords:** water on the road, road surface state, acoustics monitoring, sound intensity, smart city sensors

## Abstract

This paper presents a new approach to detecting the presence of water on a road surface, employing an acoustic vector sensor. The proposed method is based on sound intensity analysis in the frequency domain. Acoustic events, representing road vehicles, are detected in the sound intensity signals. The direction of the incoming sound is calculated for the individual spectral components of the intensity signal, and the components not originating from the observed road section are discarded. Next, an estimate of the road surface state is calculated from the sound intensity spectrum, and the wet surface detection is performed by comparing the estimate with a threshold. The proposed method was evaluated using sound recordings made in a real-world scenario, and the algorithm results were compared with data from a reference device. The proposed algorithm achieved 89% precision, recall and F_1_ score, and it outperforms the traditional approach based on sound pressure analysis. The test results confirm that the proposed method may be used for the detection of water on the road surface with acoustic sensors as an element of a smart city monitoring system.

## 1. Introduction

Smart city systems are the current trend in environmental monitoring. A network of sensors installed in the urban area provides continuous streams of data that are analyzed in the data centers. Monitoring of the urban traffic system is one of the most important applications of the smart city systems. The goal is to manage the city traffic efficiently and to increase the safety level for drivers and pedestrians. The presence of a water layer on the road surface is an important factor in increasing the risk of traffic accidents [1,2]. Detection of water on the road is, therefore, an essential function of a smart city system, which may alert the drivers and ask them to drive carefully. An efficient smart city system requires a large network of preferably low-cost sensors [3]. State-of-art sensors for measurement of the thickness of the water layer on the road surface are mostly based on optical (laser) sensors. Such devices are large and expensive; therefore, they are not suitable for large smart city systems.

The authors propose to use a small, low-cost acoustic sensor for the assessment of water presence on the road. Acoustic sensors are typically used in smart city systems for noise level measurements [4], but they are capable of providing other useful data. In the previous work, the authors evaluated the usefulness of an acoustic vector sensor (AVS) in traffic analysis. Contrary to standard, single microphone sensors that only measure sound pressure, the AVS measures sound intensity, which is a vector quantity and allows for the determination of the incoming sound direction [5,6]. In previous publications, the authors have successfully applied the sound intensity analysis to the detection of road vehicles and their direction of movement [7]. This paper presents a new application of the sound intensity analysis for the determination of the water presence on the road surface. Preliminary results were presented during the conference talk [8]. Here, a fully developed method is presented, together with the test results, compared with data obtained from the reference device.

Published works on the detection of water on a road using acoustic methods are based on pressure signal analysis. Most of the published methods utilize a machine learning approach, which, while effective, requires a large set of training examples. Abdic et al. [9] used a recurrent Neural Network (NN) trained on 785,826 audio examples and achieved 93.2% recall. Kongrattanaprasert et al. [10] utilized multiple NNs with the learning vector quantization networks and obtained 80% accuracy. Shariff et al. [11] employed convolutional NN with transfer learning to avoid the need for supervised learning with a large dataset, achieving above 80% accuracy. In another publication [12], they trained an NN with scalograms calculated from audio data and reached almost 90% accuracy. Bahrami et al. [13] used a two-stream convolutional NN for the analysis of acoustic features extracted from the noise recorded inside the vehicle. Alonso et al. [14] proposed a method suitable for on-board analysis of tire noise combined with data from the vehicle microcomputer using Support Vector Machines. Kalliris et al. [15] compared various machine learning algorithms and found that Support Vector Machines using Quadratic and Cubic kernels achieved the best results. Wang et al. [16] used an NN trained with a combined set of features extracted from both images and the tire noise, resulting in 92.7% accuracy. Akama et al. [17] used a different approach based on a Bayesian estimator, reporting 99% accuracy in their test set.

The published works mentioned above used the traditional approach based on the analysis of pressure signals recorded with single or multiple microphones. Analysis of sound intensity provides additional information on the sound source direction without the need to use complex microphone setups and apply beamforming algorithms. Sound intensity probes have been known for many years, but due to their high cost compared with standard microphones, they were rarely considered for applications such as the one described in this paper. Acoustic vector sensors built from pairs of matched microphones are also known, but they were rarely used in research, mostly because of the large sensor size when standard microphones are used. Advances in the technology resulted in the appearance of miniature (less than 5 mm) MEMS (Micro-Electro-Mechanical System) microphones. With these microphones, it became possible to construct a small AVS able to determine sound intensity and sound source direction. The aim of this paper is to propose the following modifications to the methods of detection of water on the road surface published in the literature so far. The analysis is performed on the sound intensity instead of the sound pressure, which makes it possible to determine the direction of sound sources. Using this information, the proposed method attenuates sound intensity components not originating from the vehicle tires making contact with the surface of an observed road section. The proposed modifications allow for more accurate sound analysis than in the case of pressure sensors.

The rest of the paper is organized as follows. The stages of the method are described: sound intensity calculation, determination of the incoming sound direction, elimination of the unwanted signal components, detection of acoustic events and, finally, calculation of the measure that represents the presence of the water layer on the road. The next chapter describes the test setup, the results of the performed experiments, the comparison with the reference data and the discussion. The paper ends with conclusions and indications of future work.

## 2. Materials and Methods

**Figure 1:**
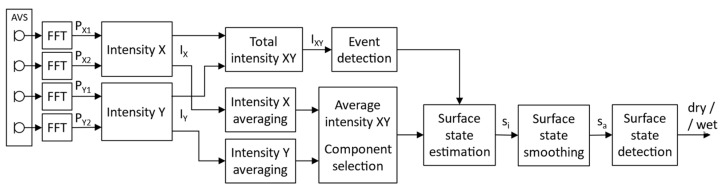
Block diagram of the algorithm.

The proposed method of detecting water presence on the road surface using an AVS works in several stages. A general block diagram is shown in Figure 1, and the details of each stage are presented in the following subsections of the paper.

### 2.1. Sound Intensity

Sound intensity is a measure that describes the energy flow in sound waves, defined as the power carried by sound waves per unit area in a direction perpendicular to that area. Sound intensity, methods of measurement and practical applications became known to the acousticians thanks to the research published by Fahy [18], later extended by Jacobsen [19], with a focus on the measurement methods. Sound intensity is a vector quantity defined as:

(1) $$I=\frac{1}{T}\overset{T}{\underset{0}{\int }}p\left(t\right)u\left(t\right)dt,$$

where *p*(*t*) is sound pressure (scalar), and **u**(*t*) is acoustic velocity (vector).

The acoustic velocity **u** may be approximated with a pressure gradient calculated from the measurement obtained from two closely spaced microphones: *p*_1_(*t*), *p*_2_(*t*). This is called a ‘p-p’ method, and it requires that the two microphones are matched in terms of their parameters—this may be ensured using a calibration procedure [20]. Instantaneous sound intensity may then be calculated as [18]:

(2) $$I\left(t\right)=\frac{p_{1}\left(t\right)+p_{2}\left(t\right)}{2\rho r}\overset{t}{\underset{-\infty }{\int }}\left(p_{1}\left(t\right)-p_{2}\left(t\right)\right)dt,$$

where *ρ* is air density, and *r* is the spacing between the pressure sensors.

Sound intensity may be calculated in the time domain by averaging the instantaneous intensity or in the frequency domain. The proposed method uses the latter approach. Sound intensity is calculated using the formula [21]:

(3) $$I\left(\omega \right)=\frac{1}{2\rho r\omega }Im\left(P_{1}\cdot P_{2}^{*}\right),$$

where *P_i_* is the Fourier transform of the pressure *p_i_*, *Im* is the imaginary part of the complex spectrum, asterisk denotes the complex conjugation, and ω is the angular frequency. The advantage of the spectral approach is that sound intensity may be calculated for each spectral component independently.

### 2.2. Selection of Intensity Components Based on Their Direction

**Figure 2:**
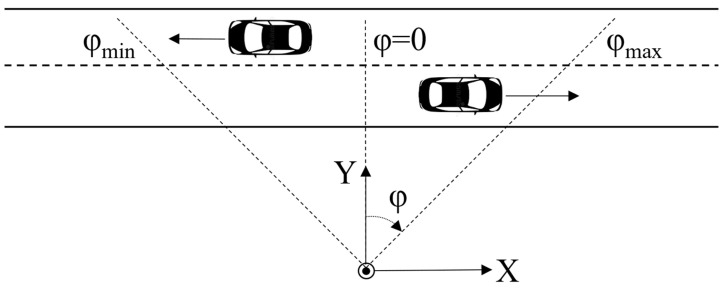
Sensor coordinate system relative to the road.

Sound intensity calculated from pressure measured by two microphones represents the flow of acoustic energy along the axis determined by the two sensors. If two identical pairs of omnidirectional microphones are placed on the orthogonal axes so that the middle points of both pairs are at the same location, a two-dimensional acoustic vector sensor (2D AVS) is obtained. Such a sensor is able to determine the azimuth *φ* of a sound source using sound intensities *I_X_*, *I_Y_* measured along the *X-* and *Y*-axes, respectively (Figure 2):

(4) $$\varphi =arctan\left(\frac{I_{X}}{I_{Y}}\right).$$

If sound intensity is calculated in the frequency domain, the sound source azimuth can be calculated for the individual spectral components:

(5) $$\varphi_{k}=arctan\left(\frac{I_{X,k}}{I_{Y,k}}\right),$$

where *k* is the spectral bin index with central frequency *f_k_* given by:

(6) $$f_{k}=k\frac{f_{s}}{K},$$

where *f_s_* is the sampling frequency, and *K* is the Fourier transform length (in this paper, sound intensity analysis is performed in the digital domain).

With the above equations, an “azimuth spectrum” may be computed, providing information on the sound source direction on a time-frequency plane. Total sound intensity may be calculated by averaging the frequency components within a frequency range defined by the bin indices $\langle k_{min},\;k_{max}\rangle$:

(7) $$I=\frac{1}{k_{max}-k_{min}+1}\overset{k_{max}}{\underset{k=k_{min}}{\sum }}I_{k},$$

where *I_k_* is the *k*-th bin of the sound intensity spectrum. This way, sound intensity limited to a specified frequency range can be calculated. The whole frequency range can also be divided into bands, and sound intensity in each band can be easily computed.

Another advantage of the approach presented here is that spectral components of the sound intensity signal may be selected according to their azimuth. Each spectral component is represented with a pair (*I_k_*, *φ_k_*). Therefore, the summation in Equation (7) may be supplemented with a condition:

(8) $$I=\frac{1}{k_{max}-k_{min}+1}\underset{k\in B}{\sum }I_{k},$$

where:

(9) $$B=\left\{k\in \left\{k_{min},\ldots ,k_{max}\right\}\left|\varphi_{k}\geq \varphi_{min}\right.\wedge \varphi_{k}\leq \varphi_{max}\right\}.$$

where the range of azimuth values that are of interest is defined by (*φ*_min_, *φ*_max_).

In practical situations, if the sensor is positioned so that the zero azimuth corresponds to the axis perpendicular to the road (Figure 2), sounds originating from the vehicle moving on the road will be limited to a specific azimuth range, for example, −20° to 20°. By limiting the sound intensity analysis to components originating from directions within the range of interest, it is possible to reduce the level of the unwanted sounds from the environment, thus increasing the signal-to-noise ratio for the sound intensity analysis.

### 2.3. Preliminary Results

**Figure 3:**
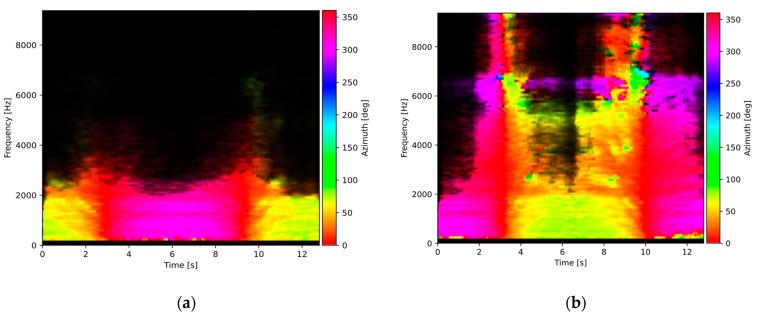
Sound source azimuth plots for: (**a**) dry road surface, (**b**) wet road surface. Pixel brightness represents the sound intensity level.

Figure 3 shows the “azimuth spectrograms” calculated for two cases: the dry and the wet road surface. Each plot was calculated for two vehicles moving in the opposite direction. The *X*-axis represents time; the *Y*-axis—frequency (limited to 9 kHz in the plots, as there are no important signal components above that frequency). Hue represents the sound source azimuth, and pixel brightness represents the sound intensity level. It can be clearly observed that in the case of a wet surface, there is a significant increase in the sound intensity for frequencies above 2.5 kHz compared to the dry surface state. However, the plots are difficult to interpret because of the presence of components with azimuth beyond the range of interest.

**Figure 4:**
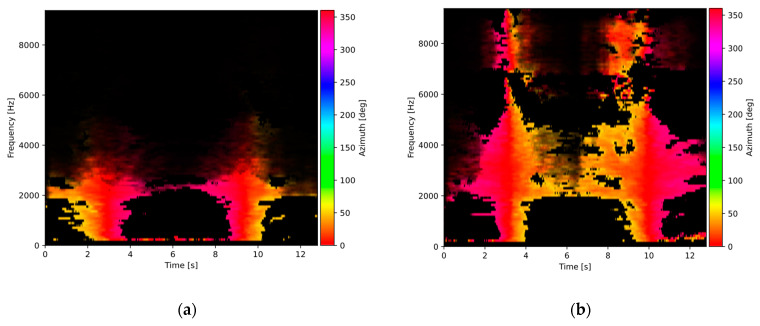
Sound source azimuth plots for: (**a**) dry road surface, (**b**) wet road surface, with components limited to the azimuth range of interest (−40° to 40°). Pixel brightness represents the sound intensity level.

Figure 4 shows the same two cases, but the azimuth components within the range of 40 to 320 degrees were removed, leaving only the components with the azimuth values corresponding to the observed road segment. The two vehicles may now be clearly seen in the plot. Removal of the unwanted signal components allows the analysis to focus on signals produced by the vehicles near the sensor and to reduce the influence of other sound sources on the analysis results.

### 2.4. Acoustic Events Detection

Road surface state should be analyzed only if the sensor receives sounds originating from vehicles moving through the observed road section. The event detection procedure analyzes the intensity signals obtained from the sensor and extracts signal parts that likely contain vehicle sounds for further analysis. An acoustic event is defined by an increase in the sound intensity relative to the background noise level. A single acoustic event represents one or more sound sources, in this case, moving vehicles. The proposed algorithm does not require dividing an event into individual moving sound sources.

It is assumed that the sensor is oriented relative to the observed road as follows: the *X*-axis of the sensor is parallel to the road, and the *Y*-axis is perpendicular to the road (Figure 2). Sound intensity is calculated according to Equation (8) by limiting the azimuth range to values corresponding to the road section, where sound intensity from moving vehicles is sufficiently higher than the noise level, e.g., −40 to 40 degrees. These values should be selected based on the distance between the sensor and the road.

Total intensity *I_XY_* in the horizontal plane (parallel to the ground) is a scalar value calculated from the intensities *I_X_*, *I_Y_* obtained from Equation (8):

(10) $$I_{XY}=\sqrt{I_{X}^{2}+I_{X}^{2}}.$$

Sound intensity calculation is limited to the spectral bin range (*k_min_*, *k_max_*) corresponding to the frequency range of approximately 400 Hz to 4 kHz, which contains the sound intensity originating from vehicle tires making contact with the road surface [22]. Lower frequencies are discarded because they contain unwanted sound intensity components from engine noise, wind, environmental noise, etc.

The calculated total intensity values *I_XY_* are smoothed with a filter to reduce the amount of noise in the analyzed signal. The presented method uses a moving average filter with an averaging time equal to c.a. 300 ms.

The intensity *I_n_* of the acoustic background (noise) is calculated using an exponential averaging filter with a long averaging time:

(11) $$I_{n}\left[k\right]=\alpha \cdot I_{n}\left[k-1\right]+\left(1-\alpha \right)\cdot I_{a}\left[k-\delta \right],$$

where *α* is the averaging factor, *k* is the sample index, and *δ* is the update delay in samples. The value of *α* is related to the averaging time, which should be sufficiently large to smooth changes in the acoustic background level. Usually, *α* is close to one (0.98 to 0.998).

Detection of an acoustic event is based on the condition:

(12) $$\bar{I_{XY}}>I_{n}+m,$$

where *m* is a constant value of a detection margin. The background noise estimate *I_n_* is updated only if the condition in Equation (12) is not fulfilled.

**Figure 5:**
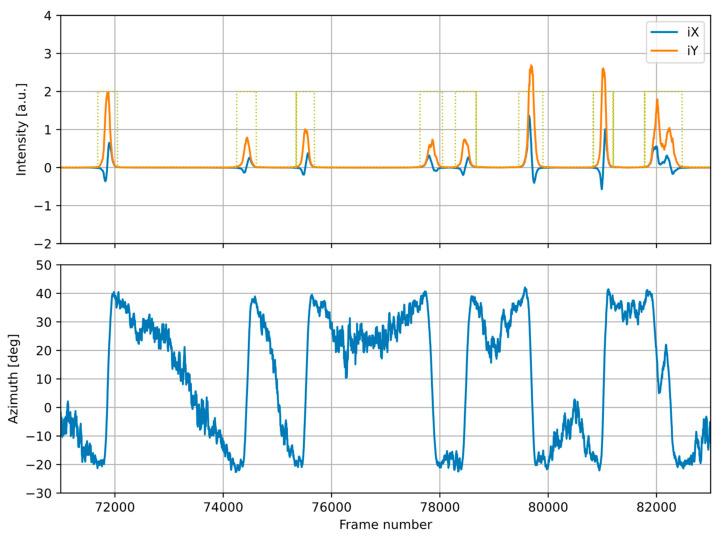
Example of the acoustic event detection: sound intensity and the detected events (**upper plot**) and the sound source azimuth (**lower plot**). Signal parts detected as acoustic events are marked with the dotted line boxes.

Figure 5 presents an example of the acoustic event detection. The upper plot shows the averaged sound intensity in two axes defining the horizontal plane. Signal parts detected as acoustic events are marked with the dotted line boxes. The bottom plot shows the sound source azimuth. It can be observed that the azimuth changes smoothly during the acoustic events, while between the events, the azimuth changes are uneven and random. The detected events in this example represent single vehicles, except for the last one (at 82,000), which contains two vehicles (two peaks in the intensity and two separate monotonic segments in the azimuth plot).

### 2.5. Estimation of the Road Surface State

**Figure 6:**
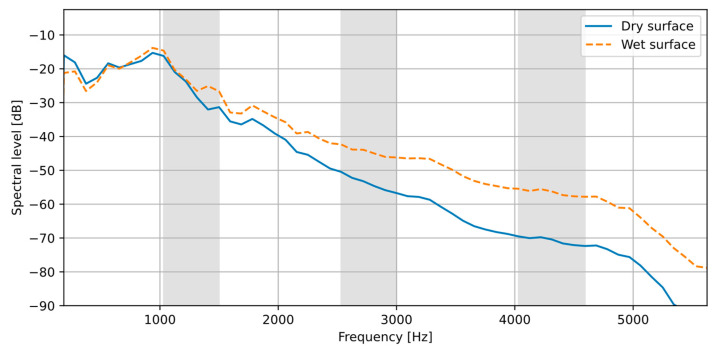
Averaged sound intensity spectra for the dry and the wet road surface. Gray regions indicate the frequency bands used for the surface state estimation.

It is shown in Figure 3 and Figure 4 that the presence of water on the road surface causes an increase in the sound intensity for frequencies above 2.5 kHz. In order to evaluate this observation more accurately, intensity spectra for all acoustic events detected in the test recordings were calculated and averaged within two classes: the dry and the wet surface, according to the data from the reference sensor. The results are shown in Figure 6. It can be observed that in the frequency range up to about 1 kHz, there is no difference in spectra for both surface types. However, as the frequency increases, the spectrum for the wet surface exhibits a noticeably higher level than the dry surface spectrum. Therefore, sound intensity in the high-frequency range acts as a discriminating factor in the surface state analysis, while the low-frequency range intensity may be used as a normalizing factor.

Based on this observation, in the proposed method, the sound intensity is calculated using Equation (8) in three separate frequency bands: *I*_1k_, *I*_3k_, *I*_4k_. For each intensity signal frame, spectral components are selected according to their azimuth, as described in the previous sections. Individual spectral components are then time-averaged using a moving average filter (in the presented case, a filter with the averaging window spanning c.a. 330 ms) to reduce the measurement noise present in the calculated sound intensity spectra. Next, the total intensity in each frequency band is calculated using Equation (8). The frequency ranges for each band and their corresponding spectral bin indices, calculated for the Fourier transform length *K* = 512, are given in Table 1.

Based on the observation regarding the difference of the average spectra for the dry and wet surfaces, the values *I*_3k_ and *I*_4k_ are used to discriminate between these two surface types, while *I*_1k_ serves as a normalizing factor. The proposed instantaneous surface state measure *s_i_*, estimated from a single acoustic event, is calculated as:

(13) $$s_{i}=\sqrt{{\left(\frac{\bar{I_{3k}}}{\bar{I_{1k}}}\right)}^{2}+{\left(\frac{\bar{I_{4k}}}{\bar{I_{1k}}}\right)}^{2}},$$

where the intensity values *I*_1k_, *I*_3k_, *I*_4k_ are averaged over the whole acoustic event.

This method of calculating *s_i_* was chosen based on the analysis of the collected data. Other approaches, such as the one based on the spectral slope, were also tested, but they were less accurate than the one presented here. In the example presented in Figure 6, *s_i_* = 0.03 for the dry surface and 0.071 for the wet surface.

Calculation of *s_i_* is valid provided that the intensity spectrum has a shape similar to the ones presented in Figure 6, i.e., with a smooth, decreasing spectral envelope above 2.5 kHz. However, it is possible that the spectrum becomes distorted, e.g., by acoustic interference in the analyzed frequency bands. Such results should be discarded from the analysis. A spectral flatness measure is used for determining the validity of *s_i_*:

(14) $$sf=\frac{exp\left(\frac{1}{k_{2}-k_{1}+1}\sum_{k=k_{1}}^{k_{2}}ln\left(L_{k}\right)\right)}{\frac{1}{k_{2}-k_{1}+1}\sum_{k=k_{1}}^{k_{2}}L_{k}},$$

where:

(15) $$L_{k}=20log_{10}\left(I_{k}\right)+100,$$

*k*_1_ and *k*_2_ are frequency bins equal to *k*_min_ of *I*_3k_ and *k*_max_ of *I*_4k_, respectively. If the spectral flatness is below the threshold (0.75 is used in the algorithm), the result is discarded.

The *s_i_* values are calculated for single acoustic events, and as such, they may be inaccurate. For example, the presence of a high-intensity acoustic distortion from the direction of the road may lead to a false positive result. However, in a typical scenario, multiple vehicles are present within an observation period. Therefore, the *s_i_* values may be time-averaged to provide an improved surface state estimate. In the proposed method, a two-stage processing is performed. The first stage is realized with a median filter with a short window (e.g., five events), the purpose of which is to remove the outliers. The second stage is a standard moving average filter with a longer window (e.g., 11 events), which reduces noise in the computed values. A longer filter window provides better noise reduction at the cost of delaying the surface state change detection. After the filtering, the averaged surface state estimate *s_a_* is obtained.

The final decision on the road surface state (dry/wet) is performed by comparing the *s_a_* values with the threshold value. In the experiments presented here, the threshold was constant, and the selection of its value is discussed further in the paper. Adaptive threshold selection was considered (a dynamic threshold set by continuously estimating the background noise level and varying the threshold value according to the current noise estimate), but due to the complexity of its implementation, it was left for future research.

## 3. Experiments

### 3.1. Test Setup

Validation of the proposed method was performed in a real-world scenario using a custom-built AVS. The sensor was constructed from six omnidirectional, digital MEMS microphones (IvenSense INMP441 [23]), with sensitivity −26 dBFS (decibels relative to full scale), providing pressure signals sampled at 48 kHz, with 24-bit resolution, using the I^2^S protocol. Microphones were mounted on the sides of a cube with an edge length of 10 mm. The microphone signals were received by a microcomputer (Raspberry Pi 4) through an I^2^S-USB interface. The signals from the sensor microphones were recorded into 15-min-long files (six channels of uncompressed pressure data) and stored on a flash drive.

**Figure 7:**
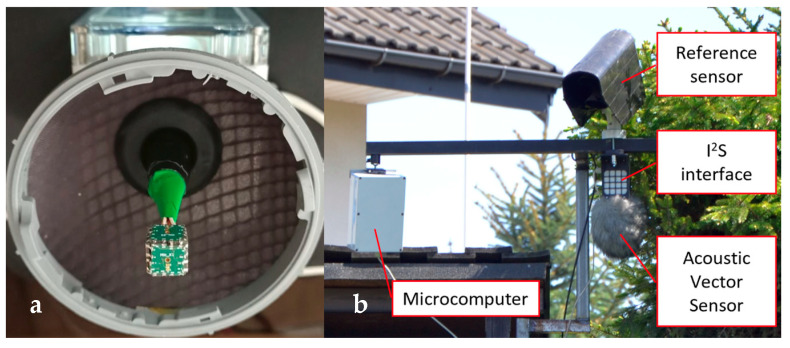
The AVS (**a**) and the test setup (**b**) used for the evaluation of the proposed method.

The test setup was installed in a rural area by the side of a straight section of a busy regional road with an even asphalt surface. The sensor was placed in a protective enclosure connected to a box containing the microcomputer and the power source (Figure 7). The sensor was mounted c.a. 7.2 m away from the road edge at a height of 4.3 m.

A remote road surface state sensor Vaisala DSC111 [24], mounted above the AVS, was used as a reference device. This is a professional, certified device that measures the thickness of the water layer on the road using a spectroscopic sensor. Data from the reference sensor were recorded in the 120 s intervals, and they constitute the ground truth data for the proposed method evaluation.

The data were collected during July 2023. The AVS recordings from nine non-consecutive days were selected for the analysis based on the presence of water on the road surface reported by the reference sensor. For each of the nine days, continuous 24-h recordings were analyzed. A total of 216 h of recordings were analyzed. The recorded signals were processed offline on a computer using Matlab and Python scripts. The detection algorithm was also implemented using Python scripts so that it can be used in the online mode, processing live signals and outputting the results.

The AVS was calibrated in an anechoic chamber by measuring impulse responses from each microphone. A correction function was calculated in the frequency domain for each microphone to equalize differences between microphones on each axis [20]. These correction functions were applied to the pressure signal spectra before the intensity signals were calculated.

For the detection of acoustic events, the analysis was limited to the frequency range 375 Hz to 4031.25 Hz, and the spectral components corresponding to the azimuth range −30 to 50 degrees were selected (the sensor was rotated by c.a. 10 degrees relative to the road). During the surface state estimation, the azimuth range was further limited to −10 to 30 degrees (the range was selected based on the distance of the sensor from the road). The signals were analyzed in blocks of 512 samples at 48 kHz sampling frequency (block length 10.67 ms). The averaging filter length for the event detection and the intensity spectrum smoothing was 31 blocks (330.67 ms). The instantaneous surface state estimates were processed by the median filter of length 5 samples, then by the moving average filter with length 11 samples.

Short time frames of 512 samples were used to achieve good temporal resolution (10.67 ms) so that the acoustic events are analyzed as soon as possible. The frame length affects the frequency resolution during the spectral analysis of the intensity signals—it is equal to 93.75 Hz. The experiments proved that such frequency resolution is sufficient for the proposed algorithm. Frequency resolution may be improved by increasing the time frame at the cost of reduced temporal resolution.

### 3.2. Results

A total of 24,170 acoustic events were detected in the test set, which means that, on average, about 112 events were detected during one hour of recordings (one event may contain multiple vehicles). The detected events were recorded as timestamped values of the averaged surface state estimates *s_a_*. The ground truth data consisted of the water layer thickness measured by the reference sensor at 120 s intervals. In order to match these two datasets, the algorithm results *s_a_* were resampled to the time intervals defined by the reference data using linear interpolation. The binary decision wet/dry was performed by comparing these values with a threshold. For the reference data, a threshold of the water layer thickness equal to 0.2 mm was used (according to the data recorded by the reference sensor, this threshold separates the “dry” and “wet” surface state classes). In the reference sensor data, 7.14% of readouts indicated a wet surface.

**Figure 8:**
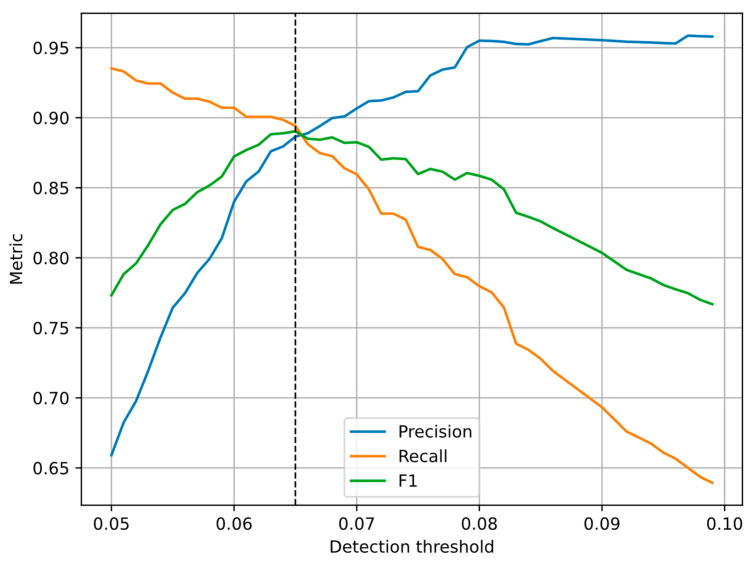
RoC curves for different detection thresholds in the evaluated algorithm. The vertical line shows the value of equal precision and recall.

The choice of the detection threshold for the evaluated algorithm was made by calculating the accuracy metrics (precision, recall and F1-score) for different threshold values using the whole dataset. The resulting RoC (receiver operating characteristics) curves are shown in Figure 8. A threshold of 0.065 provided equal precision and recall values, and it was chosen as the decision threshold for the experiments. A different threshold value may be used if a higher precision (less false negative results) or a higher recall (less false positive results) is preferred.

Table 2 shows the results obtained from the analysis of the whole dataset using the proposed method. The main version of the evaluated algorithm (Alg. 1) is the proposed method based on sound intensity analysis, including the selection of spectral components with azimuth covering the observed road section. Two other approaches were evaluated for comparison. The purpose of these two algorithms is to evaluate the advantage of using the sound intensity analysis and the selection of spectral components based on the azimuth, as implemented in Alg. 1. The first method (Alg. 2) is also based on the sound intensity, but all spectral components are considered, regardless of their azimuth. The other method (Alg. 3) is the standard approach based on the sound pressure (signals from a single microphone) instead of the sound intensity. With this approach, determining the source azimuth is not possible. For each case, the threshold value was found using the procedure described earlier and rounded to the third decimal place. The results are discussed in the next section.

**Figure 9:**
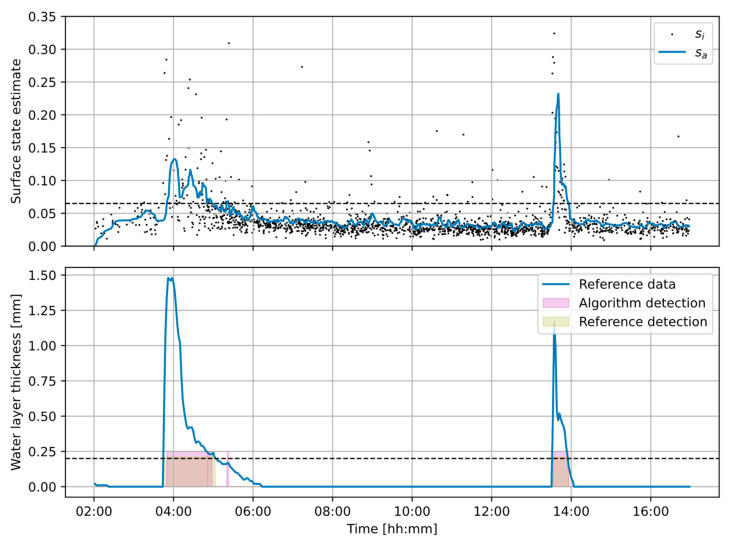
Example of the analysis results, with two periods of rainfall. (**Upper plot**): surface state estimates *s_i_* and *s_a_*. (**Bottom plot**): reference data and the “wet surface” detection results. Dashed horizontal lines indicate the thresholds.

An example of the detection results and comparison with the reference data is presented in Figure 9. The upper plot shows the surface state estimates calculated by the algorithm: dots present the instantaneous values *s_i_* for the individual acoustic events; the line shows the averaged *s_a_* values. The bottom plot line shows the data from the reference sensor. Signal sections indicating wet surface (values above the threshold) are marked with colored boxes: purple for the algorithm detection and light brown for the reference data detection. Areas marked with dark brown color indicate parts where the wet surface was detected by both the algorithm and the reference sensor.

## 4. Discussion

The detection threshold in the algorithm was selected so that the precision and the recall are approximately equal, which also means that the F_1_ score is the same. Hence, the term “accuracy” will be used to describe all three metrics. The proposed algorithm achieved c.a. 89% accuracy in the detection of the presence of water on the road surface when compared with the data from the reference sensor. Given the complexity of the problem of estimating the road surface state using only audio signal analysis, that level of accuracy may be considered satisfactory. It should be noted that the surface state was evaluated at 120 s intervals. A false negative result does not mean that, e.g., rainfall was completely missed. Many of the false negative and false positive detection results were caused by the detection delay, i.e., the detector changing its state too late. Similarly, there were some false positive results that lasted only for a short time. It should also be noted that the reference sensor measures the water layer at one specific point on the road, while the proposed method analyzes a larger section of the road. This aspect may influence the results comparison after a rainfall, especially if the road surface is uneven or it is not uniformly covered by sunlight.

The choice of the detection threshold for the evaluated algorithm was based on the equal precision and recall condition. In a real-life application, the threshold should be tunable so that a desired balance between the precision and the accuracy may be obtained. As expected, increasing the threshold improves the precision, reducing the risk of false negative results, but at the same time, it deteriorates the recall, increasing the risk of false positive results. Decreasing the threshold has the opposite effect. In a practical installation, reducing the risk of false negative results may be preferred so that the threshold value may be increased. It should also be noted that the proposed method achieves above 76% F_1_ score for the whole range of the tested threshold values 0.05 to 0.10 (Figure 8).

A plot of example results in Figure 9 shows that although the majority of the instantaneous surface state estimates *s_i_* follow the surface state changes (their values increase as the surface becomes wet), there are some results that deviate from the trend. This is most likely caused by acoustic sources in vehicles that emit sounds not related to the tires in the analyzed frequency range, increasing the spectral intensity level and causing higher *s_i_* values on a dry surface. Such cases occur mostly for larger vehicles (e.g., trucks) but only for some of them. The proposed procedure relies on the smoothing algorithm that filters out such cases from the results. Therefore, the algorithm works on the assumption that there is a sufficiently large number of the analyzed events available so that any result that deviates from the trend is discarded. This condition was fulfilled in the test setup. However, in case of very low traffic (e.g., one vehicle every five minutes), such result averaging is not possible, and the algorithm accuracy is expected to deteriorate. Figure 9 shows that water detection is more problematic during the night hours when the number of vehicles is significantly lower than during the day. Hence, in a practical application, a minimum number of events per observation period should be imposed, and the results obtained for a low number of events should not be reported. Additionally, the proposed algorithm provides a form of a “reliability” measure for the results by computing the standard deviation of the averaged instantaneous values.

From the analysis of the results plot (Figure 9), it should also be observed that the dispersion of the instantaneous values is significantly higher for the wet surface than for the dry surface. This means that although there is an increase in the intensity level for higher frequencies on the wet surface, as shown in Figure 6, the degree of the increase may depend on the vehicle size and weight, tire size and condition, etc. Therefore, the requirement of having a sufficiently large number of events for the analysis is even more important for a wet surface.

The filtering (smoothing) procedure is necessary to obtain the surface state values *s_a_* suitable for the detection. Every online filtering procedure introduces a delay to the results. If the smoothing filter length is increased, a higher level of noise suppression is obtained, making the detection easier, but at the same time, it increases the detection delay. Such a delay is unwanted in the wet surface detection; a wet surface is expected to be reported as soon as possible. Therefore, relatively short filters were used in the experiments (a median filter of length 5 and a moving average filter of length 11) as a compromise. In practical applications, the filter length may be made tunable as a “detection latency” parameter.

An important feature of the method based on sound intensity measured by an AVS is the ability to determine the azimuth (source direction) for every spectral component and to select only the components with the azimuth of the observed road section. If that function is omitted and the whole sound intensity spectrum is analyzed, the detection accuracy decreases by about 0.07 (Alg. 2 in Table 2). This result proves that the proposed method that limits the sound intensity analysis to the azimuth range of interest provides a significant increase in the wet surface detection accuracy.

A comparison of the proposed method with a similar algorithm operating on the sound pressure signals, recorded with a single microphone in the AVS (Alg. 3 in Table 2), indicates that the method based on the sound intensity with component selection has significantly higher accuracy by c.a. 0.13 than the pressure-based approach. Even if the component selection is omitted, the method based on sound intensity has an accuracy higher by c.a. 0.06 than the method based on pressure. Therefore, the road surface state estimation based on the sound intensity analysis provides significantly higher accuracy than the traditional state-of-the-art approach in which pressure signals from a single microphone are analyzed.

In the presented experiment, the sensor was analyzing traffic on a road with a single lane in each direction. The number of lanes and their direction are not important for the proposed method. The sound intensity decreases with the distance from the sensor. If a lane is too far from the sensor, the sound intensity becomes comparable with the noise level, and detection of the acoustic events is impossible. Therefore, positioning the sensor close to the road is preferred.

## 5. Conclusions

The proposed method of the detection of water on the road surface is built upon an observation that the presence of a water layer on the road changes the soundscape of the tire noise by increasing the sound intensity level in the frequency range above 1 kHz. The results of the experiments performed using the real-world recordings indicate that the proposed method has sufficient accuracy to be considered for practical applications, such as smart city systems, in which high accuracy, certified sensors are not required. Compared with the reference sensor used in the experiments, an AVS may be realized as a low-cost, small and power-efficient device suitable for installation in multiple locations within a distributed monitoring system. The algorithm can be run in quasi-real time on a microcomputer with moderate processing power. To perform an accurate detection of the water layer on the road, a sufficient level of traffic intensity is required. The sensor used by the proposed method may also provide other important data related to traffic monitoring. From the event detection results calculated with the method described here, it is possible to obtain data on traffic intensity (coverage of the observation period with the detected events). Analysis of the sound intensity and the source azimuth may also be used for vehicle detection and counting.

The experiments described in this paper were conducted to validate the proposed method. Only one specific test installation was available for the experiments. The proposed method should be tested further in other locations with different types of road surfaces, different traffic intensities, different seasons, etc. Such experiments are planned for the next stage of the research. It is expected that different conditions will require retuning of the algorithm parameters, mostly the detection threshold. One possible enhancement of the proposed algorithm is the addition of an automatic detection threshold selection based on the analysis of the noise level in the surface state estimates. This is a complex problem which requires separate research. Nevertheless, the results obtained from the test installation prove the validity of the proposed approach to the estimation of water presence on the road surface using only acoustic signals.

In this paper, an algorithmic approach to the problem was proposed. Certainly, the machine learning approach may also be considered. The sound intensity signals may be calculated as proposed here, and the average sound intensity spectrum, after the component selection based on their azimuth, may form an input vector for the machine learning algorithm, which replaces the surface state estimate calculation and the threshold decision. This approach requires collecting a much larger dataset than the one used in this paper. The authors plan to explore this method in future research. However, the algorithmic approach presented in this paper is simple, does not require high computing power, and provides good detection accuracy.

## Figures and Tables

**Table 1:** Frequency ranges used for the surface state analysis and spectral bin ranges *k* calculated for *K* = 512 (sampling rate 48 kHz).

Band	*f*_min_ [Hz]	*f*_max_ [Hz]	*k*_min_	*k*_max_
*I*_1k_	1125	1500	12	16
*I*_3k_	2625	3000	28	32
*I*_4k_	4125	4593	44	49

**Table 2:** Results of the surface state estimation using three versions of the algorithm.

Parameter	Alg. 1	Alg. 2	Alg. 3
Analyzed signals	intensity	intensity	pressure
Component selection by azimuth	yes	no	no
Detection threshold	0.065	0.053	0.207
Number of the analyzed time points	6480	6480	6480
Number of “wet surface” time points	463	463	463
True positive results (TP)	414	382	351
False negative results (FN)	49	81	112
False positive results (FP)	53	83	113
Precision: P = TP/(TP + FP)	88.6%	82.1%	75.6%
Recall: R = TP/(TP + FN)	89.4%	82.5%	75.8%
F_1_ score: F_1_ = (2 · P · R)/(P + R)	89.0%	82.3%	75.7%

## Data Availability

Not applicable.
